# Clinical study of apatinib in the treatment of stage IV osteogenic sarcoma after failure of chemotherapy

**DOI:** 10.20892/j.issn.2095-3941.2019.0397

**Published:** 2020-05-15

**Authors:** Zhichao Liao, Ting Li, Chao Zhang, Xinyue Liu, Ruwei Xing, Sheng Teng, Yun Yang, Gang Zhao, Xu Bai, Jun Zhao, Jilong Yang

**Affiliations:** ^1^Department of Bone and Soft Tissue Tumor, Tianjin Medical University Cancer Institute and Hospital; National Clinical Research Center for Cancer; Key Laboratory of Cancer Prevention and Therapy, Tianjin; Tianjin’s Clinical Research Center for Cancer, Tianjin 300060, China; ^2^Department of Pathology, Tianjin Medical University Cancer Institute and Hospital; National Clinical Research Center for Cancer; Key Laboratory of Cancer Prevention and Therapy, Tianjin; Tianjin’s Clinical Research Center for Cancer, Tianjin 300060, China; ^3^Department of Radiology, Tianjin Medical University Cancer Institute and Hospital; National Clinical Research Center for Cancer; Key Laboratory of Cancer Prevention and Therapy, Tianjin; Tianjin’s Clinical Research Center for Cancer, Tianjin 300060, China

**Keywords:** Apatinib, osteogenic sarcoma, progression-free survival, safety

## Abstract

**Objective:** To analyze the efficacy and safety of apatinib in the treatment of stage IV osteogenic sarcoma after chemotherapy failure through a single-arm, prospective, and open clinical phase II study.

**Methods:** Information on 34 patients with stage IV osteogenic sarcoma treated with apatinib after failure of chemotherapy in Tianjin Medical University Cancer Institute and Hospital between September 2015 and December 2019 was collected and analyzed. The participants included 23 males and 11 females, with an average age of 35.24 years (11–73 years). The objective response rate (ORR), disease control rate (DCR), progression-free survival (PFS), PFS rate (PFR), and overall survival (OS) were evaluated. The treatment-related adverse events (AEs) and safety of apatinib were also evaluated.

**Results:** Of the 34 patients, 33 were able to be evaluated for efficacy. One patient received apatinib treatment for less than one cycle; therefore, only safety analysis was performed. The 12-week clinical evaluation showed that 2 patients had a partial response (PR), 24 patients had stable disease (SD), and 7 patients had progressive disease (PD). The ORR, DCR, and PFR at 12 weeks were 6.06% (2/33), 78.79% (26/33), and 82%, respectively. By the end of the follow-up, 6 patients had SD (18.18%, 6/33), 27 patients had PD (81.82%, 27/33), and 15 patients died because of disease progression (45.45%, 15/33). The ORR was 0 (0/33), the DCR was 18.18% (6/33), and the median PFS (mPFS) was 7.89 months (95% CI: 4.56–11.21). The median OS (mOS) was 17.61 months (95% CI: 10.85–24.37). The most common treatment-related AEs were hand-foot syndrome (35.29%, 12/34), proteinuria (32.35%, 11/34), and hypertension (32.35%, 11/34).

**Conclusions:** Apatinib is effective and well tolerated in stage IV osteogenic sarcoma patients after chemotherapy failure.

## Introduction

Osteogenic sarcoma has an incidence of approximately 1 or 2 per 100,000 individuals, accounting for approximately 1% of all adult malignancies and 15% of all pediatric malignancies. According to the National Office for Cancer Prevention and Control, National Cancer Center, in China in 2015, 28,000 patients newly developed osteogenic sarcoma, and 20,700 died of this disease^1,2^. The common pathological types include osteosarcoma, chondrosarcoma, Ewing’s sarcoma/primitive neuroectodermal tumor (EWS/PNET), and bone undifferentiated pleomorphic sarcoma (UPS). Patients with osteogenic sarcoma and distant metastases have an extremely poor prognosis, with a median overall survival (mOS) and 5-year overall survival rate (OSR) of approximately 12 months and < 10%, respectively^3^. In particular, non-metastatic osteogenic sarcoma, such as osteosarcoma, shows a relatively good prognosis^4^, but patients whose disease relapses after failure of standard chemotherapy have poor survival, with a 5-year post-relapse survival rate below 30%, thus presenting a challenging treatment dilemma^5^. Although chemotherapy is widely used for these metastatic lesions, they are incurable with conventional chemotherapeutics such as ifosfamide, adriamycin, methotrexate, cisplatin, gemcitabine, and paclitaxel^6^. Moreover, combination therapy or high-dose, multicycle chemotherapy does not improve the response rate. In addition, large doses of chemotherapeutics also produce substantial toxic or side effects during tumor treatment^7^. For example, a high cumulative or extreme dose of adriamycin can cause cardiomyopathy, which is life-threatening^8,9^. Some patients, through aggressive surgical resection of all gross lesions, may achieve long-term survival^10^. Several clinical trials on advanced bone sarcoma are currently in progress, but there have been no breakthroughs in treatment, and the overall prognosis of advanced bone sarcoma remains poor (**Table 1**)^11–17^. Therefore, there is an urgent need to identify new treatments to improve the outcomes of these patients.

Angiogenesis is a key factor in tumor growth and metastasis, and anti-angiogenic therapy is an important method for modern cancer treatment^18^. Mesylate apatinib (YN968D1) is a novel oral micromolecular tyrosine kinase inhibitor that inhibits vascular endothelial growth factor receptor-2 (VEGFR-2), mainly through competitively binding the intracellular tyrosine in the adenosine triphosphate binding site of the receptor, thus highly selectively inhibiting the activity of the VEGFR-2 tyrosine kinase and blocking signal transduction after binding of vascular endothelial growth factor (VEGF), thereby potently inhibiting tumor angiogenesis and decreasing the density of microvessels in tumors^19–21^. Phase I–III clinical trials of apatinib have shown definite antitumor activity and controllable side effects in patients with gastric cancer, breast cancer, and non-small cell lung cancer^19,22–24^. Case reports and retrospective case analyses of apatinib in the treatment of metastatic sarcoma have shown encouraging results^25–28^. Three retrospective studies have been conducted to date on apatinib in the treatment of sarcoma, the earliest of which was completed by our oncology center^27,29,30^. These results suggest that apatinib might be a promising treatment for patients with metastatic sarcoma. To provide clinical insight into real-world practice, we registered a clinical study of osteogenic sarcoma in our hospital. In this study, we examined real-world experience in apatinib therapy for this diverse group of malignancies. We reviewed and analyzed the response patterns of 34 patients with different histologic subtypes of bone sarcoma, and our results may aid in osteogenic sarcoma treatment in clinical practice.

## Materials and methods

### Patients

We collected and analyzed the data of 34 patients with stage IV osteogenic sarcoma who received apatinib after chemotherapy failure at Tianjin Medical University Cancer Institute and Hospital. This clinical trial of apatinib complied with the Declaration of Helsinki and was approved by the Ethics Committee of Tianjin Medical University Cancer Institute and Hospital (Ethical batch number, E2017022). All patients signed written informed consent forms.

### Inclusion and exclusion criteria

**Figure 1:**
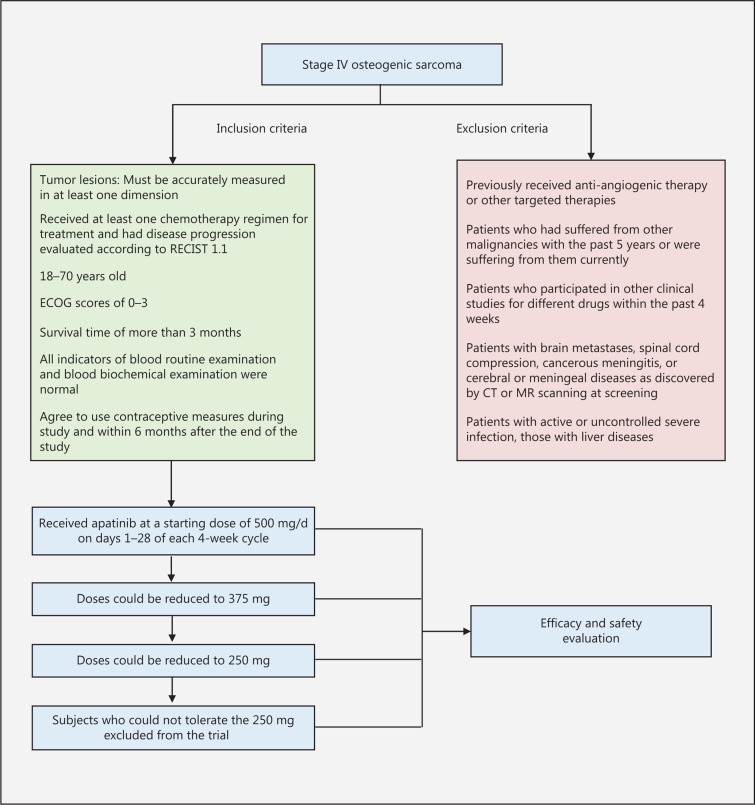
Patient inclusion and exclusion flow chart.

The inclusion criteria (**Figure 1**) were patients who volunteered to participate in the study; signed informed consent forms; had good compliance; had stage IV osteogenic sarcoma, as pathologically confirmed and clinically staged according to the TNM staging criteria of the American Joint Committee on Cancer (AJCC); had at least one lesion with measurable long and short diameters, as shown in computed tomography (CT) or magnetic resonance images; received at least one chemotherapy regimen for treatment; and had disease progression evaluated according to Response Evaluation Criteria in Solid Tumors (RECIST 1.1).

The exclusion criteria (**Figure 1**) were patients who had previously received anti-angiogenic therapy or other targeted therapies; had experienced other malignancies within the previous 5 years or currently; had participated in other clinical studies for different drugs within the previous 4 weeks; had received systemic antitumor therapy within 4 weeks before treatment or were scheduled to receive such therapy during the study; or had received extended field radiotherapy within 4 weeks before treatment or limited field radiotherapy to evaluate the tumor lesion within 2 weeks before treatment.

### Patient characteristics

From September 2015 to December 2019, we enrolled 34 patients with stage IV osteogenic sarcoma receiving apatinib in the clinical trial, including 23 males and 11 females, with an average age of 35.24 years (11–73 years) (**Table 2**). The pathological types in the 34 patients were osteosarcoma (*n* = 18), EWS/PNET (*n* = 7), bone UPS (*n* = 4), chondrosarcoma (*n* = 3), and chondroma (*n* = 2). According to the TNM staging criteria for sarcoma of AJCC, all patients had stage IV sarcoma, and the lung was the most common site of metastasis. Before treatment, most patients [55.9% (19/34)] had undergone extensive resections, and some [26.4% (9/34)] received radiotherapy.

### Treatment methods

Patients received apatinib 500 mg po qd, with warm boiled water half an hour after a meal. A duration of 28 days was considered a cycle, and the efficacy was evaluated once per cycle during the study. Patients with disease control and tolerable adverse events (AEs) used the drug continuously until continuation of the medication was deemed unsuitable by the investigator or the efficacy was evaluated as progressive disease (PD), and the safety was evaluated simultaneously during the medication course. Before PD, the patients could not receive other antitumor treatments.

AEs were closely monitored during apatinib treatment, and the dosage was adjusted as needed to make the treatment tolerable. AEs to apatinib were treated with symptomatic treatment, withdrawal, or dosage adjustment. In this clinical study, dosage adjustments occurred primarily in the second and third cycles (a duration of 28 days was considered a cycle). When a patient experienced a grade 3/4 hematologic or nonhematologic AE, the drug was withdrawn temporarily (not more than 2 weeks) until the condition was relieved or disappeared, and then the drug was taken at the original dosage. If the AEs were still not relieved after 2 weeks of drug withdrawal, the dosage was adjusted as follows: (1) first dosage adjustment: 375 mg qd; (2) second dosage adjustment: 250 mg qd. If a dosage adjustment was needed a third time, the drug was withdrawn.

### Efficacy

The objective of this study was to evaluate the efficacy and safety of apatinib in patients with stage IV osteogenic sarcoma who did not respond to chemotherapy. All patients who received at least one dose of apatinib were included in the safety and toxicity analyses, and those who had received at least one cycle of apatinib were able to be analyzed for efficacy.

#### Short-term efficacy

According to RECIST 1.1^31^, the efficacy was evaluated as complete response (CR), partial response (PR), stable disease (SD), or progressive disease (PD). The objective response rate (ORR) was calculated as (CR + PR)/total case number × 100%. The disease control rate (DCR) was calculated as (CR + PR + SD)/total case number × 100%.

#### Long-term efficacy

The progression-free survival (PFS) referred to the duration from the initiation of treatment to disease progression, and OS referred to the duration from treatment initiation to death from any cause (for patients who were lost to follow-up, it was the date of last follow-up, and for patients who were alive at the end of the study, it was the day of the end of follow-up).

#### AEs

The AEs were classified into 5 grades according to Common Terminology Criteria for Adverse Events (CTCAE) Version 5.0 of the US National Cancer Institute according to the symptoms, physical signs, and related examination records of the patients. Grade 1 included asymptomatic or mild symptoms, and clinical or diagnostic observations only, and intervention was not indicated. Grade 2 included indicated moderate, minimal, local, or noninvasive intervention, as well as limited age-appropriate instrumental activities of daily living (ADL). Grade 3 included indicated severe or medically significant but not immediately life-threatening events, hospitalization, or prolongation of hospitalization, as well as disabling or limiting self-care ADL. Grade 4 included indicated life-threatening consequences and urgent intervention. Grade 5 included death associated with the AE.

### Statistical analysis

PFS and OS were calculated with the life table method. Kaplan-Meier and Mantel-Cox tests were used to compare PFS and OS between groups. *P* < 0.05 was defined as statistically significant. All data were entered into the database and analyzed with the SPSS 20.0 statistical package.

## Results

### General results

Efficacy was evaluated according to RECIST 1.1^31,32^, the imaging data were assessed by 2 radiologists independently, and each patient had at least one measurable extracranial lesion. A total of 33 of the 34 patients with stage IV osteogenic sarcoma treated with apatinib were evaluable for efficacy, and the remaining 1 patient with chondrosarcoma took the drug orally for less than one cycle; therefore, only safety and AEs were assessed in that patient. Of the 33 patients, 31 had measurable target lesions, and 2 had unmeasurable lesions. As of December 2019, the follow-up time ranged from 2.60 to 34.37 months, with a mean of 12.78 months.

### Short-term efficacy

**Figure 2:**
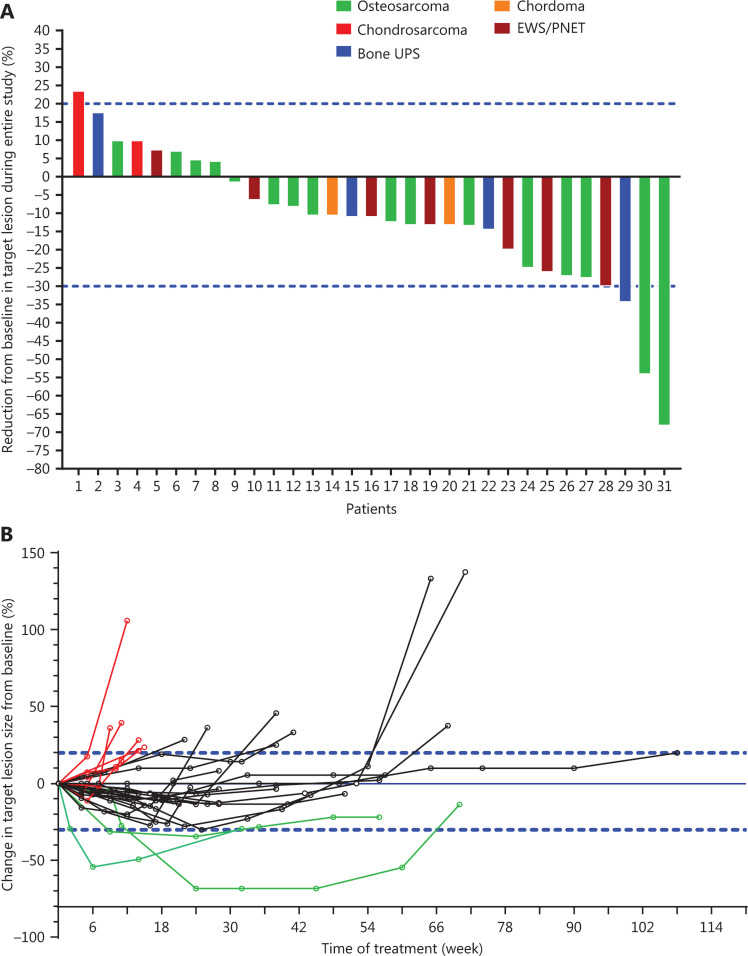
Maximum and whole-course changes in sizes of target lesions *vs*. baseline after apatinib treatment of stage IV osteogenic sarcoma that did not respond to chemotherapy. (A) Maximum changes in sizes of target lesions *vs*. baseline after apatinib treatment of stage IV osteogenic sarcoma that did not respond to chemotherapy. (B) Whole-course changes in sizes of target lesions *vs*. baseline after apatinib treatment for stage IV osteogenic sarcoma that did not respond to chemotherapy.

The maximum changes in measurable target lesions in the 31 patients are shown in **Figure 2A**. Although no patients achieved CR, only 1 (3.03%, 1/33) patient did not respond to the drug, and 32 (96.97%, 32/33) patients had initial responses to the drug. The changes in measurable lesion size during treatment compared with that at baseline are shown in **Figure 2B**.

**Figure 3:**
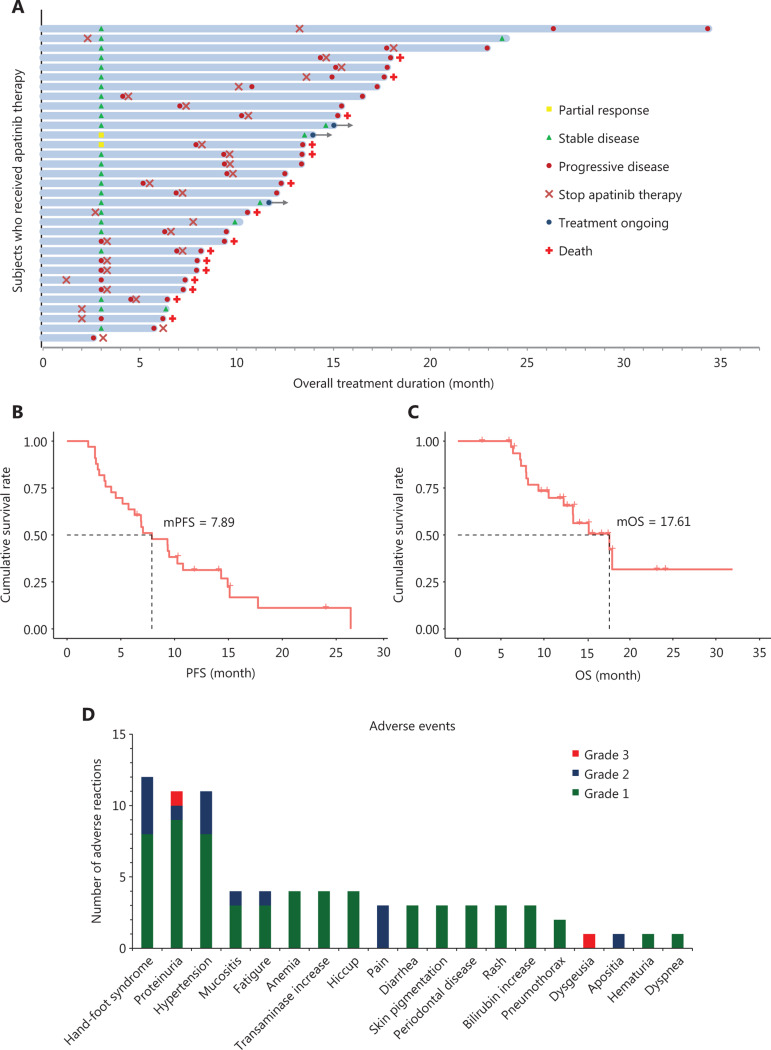
Efficacy and toxicity of apatinib in stage IV oesteogenic sarcoma. (A) Overall responses of 33 patients with stage IV osteogenic sarcoma treated with apatinib. Among 33 patients, 31 had measurable lesions, and 2 patients had unmeasurable lesions. Responses at 12 weeks were PR in 2 (6.06%, 2/33), SD in 24 (72.73%, 24/33), and PD in 7 (21.21%, 7/33). (B) PFS after apatinib treatment of stage IV osteogenic sarcoma that did not respond to chemotherapy (mPFS: 7.89 months). (C) OS after apatinib treatment of stage IV osteogenic sarcoma that did not respond to chemotherapy (mOS: 17.61 months). (D) Frequencies and grades of adverse events to apatinib.

At 12 weeks after treatment, 2 of the 33 patients were evaluated as having PR, 24 patients were evaluated as having SD, and only 7 patients were evaluated as having PD (**Table 3**, **Figure 3A**). Therefore, the ORR, DCR, and PFR at 12 weeks were 6.06% (2/33), 78.79% (26/33), and 82% (**Table 3**), respectively, thus suggesting that most patients responded to apatinib monotherapy. There was no significant difference in efficacy among patients with different types of stage IV osteogenic sarcoma (Fisher’s exact test = 0.761) (**Table 3**).

### Long-term efficacy

The mean follow-up time was 12.78 months (range: 2.60–34.37). No patient was evaluated as having CR or PR, 6 were evaluated as having SD (18.18%, 6/33), 27 were evaluated as having PD (81.82%, 27/33), and 15 died of PD (**Table 3**, **Figure 3A**). The overall DCR in the patients was only 18.18% (6/33), and there were no significant differences among patients with different types of tumors (Fisher’s exact test = 0.589) (**Table 3**). The median PFS (mPFS) was 7.89 months (95% CI: 4.56–11.21), and the median OS (mOS) was 17.61 months (95% CI: 10.85–24.37) (**Table 3**, **Figure 3B-3C**).

We also analyzed the effects of different clinical factors on the efficacy of apatinib, and found that the efficacy was not significantly affected by age (Fisher’s exact test = 1.000), ECOG score (Fisher’s exact test = 0.665), sex (Fisher’s exact test = 0.336), and local treatments before taking the drug (such as radiotherapy, Fisher’s exact test = 1.000).

**Figure 4:**
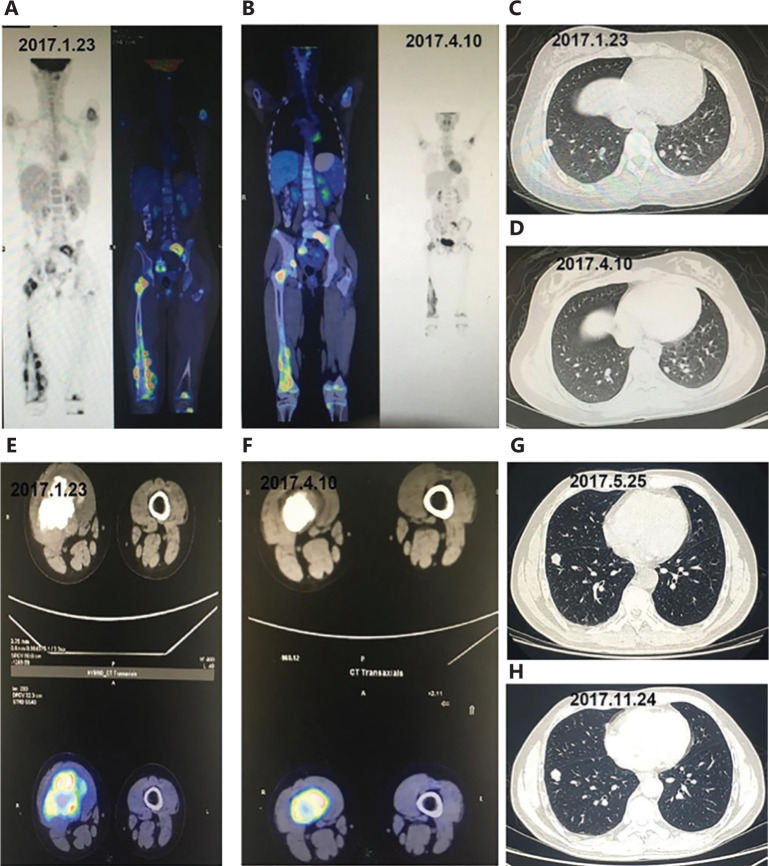
Comparison of imaging findings between 2 patients treated with apatinib for stage IV osteogenic sarcoma that did not respond to chemotherapy. (A-F) Efficacy in patients who were diagnosed with stage IV osteosarcoma at the first visit and received apatinib after they did not respond to first-line chemotherapy. (A, C, E) Imaging findings before treatment with apatinib (January 23, 2017). (B, D, F) Positron emission tomography-CT showed significant decreases in the sizes and metabolic activity of the tumors after treatment with apatinib (April 10, 2017). (G-H) Effects of apatinib in patients with metastatic Ewing’s sarcoma in the lungs who did not respond to first-line chemotherapy. (G) CT images of the chest before treatment with apatinib (May 25, 2017). (H) The lesions in the lungs remained stable from initiation of oral use of apatinib on May 25, 2017 to re-examination on November 24, 2017.

Typical patients are shown in **Figure 4**. **Figures 4A-4F** show the treatment efficacy in patients who were diagnosed with stage IV osteosarcoma at the first visit and received apatinib after they did not respond to first-line chemotherapy. Re-examination showed decreased sizes of both the primary lesions in the femur and the metastases in the lungs after 2 cycles of oral apatinib, and positron emission tomography showed significant decreases in tumor metabolic activity after treatment with apatinib. **Figures 4G-4H** show the effects of apatinib in patients with metastatic Ewing’s sarcoma in the lungs who did not respond to first-line chemotherapy. CT images of the patient’s chest showed that the lesions in the lungs were stable from the initiation of oral apatinib on May 25, 2017 to the re-examination on November 24, 2017.

### Safety and toxicity

Most AEs were mild (grades 1 to 2) and controllable (**Table 4**, **Figure 3D**). Among all drug-related AEs, grades 1, 2, 3, and 4 accounted for 79.49% (62/78), 17.95% (14/78), 2.56% (2/78), and 0 (0/78), respectively. 

A total of 5 patients had their dosages reduced during treatment because of nonhematological AEs, including hand-foot syndrome in 3 patients and proteinuria in 2 patients. One patient’s symptoms were relieved after 1 cycle of dose-reduced apatinib (250 mg/day) because of grade 3 proteinuria, and one patient stopped the trial because of grade 3 dysgeusia. Hand-foot syndrome and proteinuria were rapidly reversed and were tolerable after dosage discontinuation or reduction. Therefore, careful toxicity monitoring and timely dosage discontinuation or reduction (from 500 mg to 375 mg or 250 mg) were essential during treatment.

The most common grade 2 treatment-related AE was hand-foot syndrome (11.76%, 4/34), and the most common all-grade AEs were hand-foot syndrome (35.29%, 12/34), proteinuria (32.35%, 11/34), hypertension (32.35%, 11/34), mucositis (11.76%, 4/34), fatigue (11.76%, 4/34), anemia (11.76%, 4/34), transaminase increase (11.76%, 4/34), hiccups (11.76%, 4/34), pain (8.82%, 3/34), diarrhea (8.82%, 3/34), skin pigmentation (8.82%, 3/34), periodontal disease (8.82%, 3/34), rash (8.82%, 3/34), and elevated bilirubin (8.82%, 3/34) (**Table 4**, **Figure 3D**). For grade 2 or 3 AEs, in addition to dosage reduction and discontinuation, adjunctive treatments with related drugs were applicable. For example, patients with hand-foot syndrome were able to receive oral vitamin B_6_ and topical urea ointment, and those with hypertension were able to take angiotensin receptor antagonists or calcium antagonists orally to control the conditions. In this study, there were no serious drug-related AEs, and all AEs were significantly controlled after dosage reduction and discontinuation as well as adjuvant drug treatments, without clear sequelae or AEs with the continued application of apatinib.

## Discussion

Osteogenic sarcoma is a malignant mesenchymal tumor with unique clinical and histological features, with more than 10 subtypes^33^. Although it is not as common as other epithelial malignancies, it has a relatively high prevalence in adolescents and is the third leading cause of cancer-related death in people under 20 years of age^1,34^. Despite the dramatic improvements in multimodal therapy in recent years, the 5-year survival rate remains relatively unchanged^34^, as is particularly evident in patients with metastatic or recurrent advanced diseases. The role of second-line chemotherapy for recurrent osteosarcoma is less well defined, and there is no accepted standard regimen. Only gemcitabine-based therapy appears to have some activity in metastatic osteosarcoma^6^, Therefore, new strategies and innovative therapies are urgently needed for patients with primary malignant bone tumors.

Apatinib is an oral micromolecular tyrosine kinase inhibitor of VEGFR-2 with antiangiogenic and antitumor activity^19^. Overexpression of the VEGFR family, especially VEGFR-2, is significantly associated with a poor survival rate in patients with sarcoma^35^. Because of their effects on angiogenesis, VEGF/VEGFR targeted therapies are often used in the treatment of sarcoma. A study has shown that apatinib inhibits the growth of osteosarcoma *in vivo* and *in vitro*, and induces the autophagy and apoptosis of osteosarcoma cells by directly inhibiting the expression of the anti-apoptotic protein Bcl-2 and inactivating signal transduction and activator of transcription 3 (STAT3)^35^. Another study has shown that apatinib weakens migration and invasion by inhibiting epithelial-mesenchymal transition and inactivating STAT3; in addition, apatinib decreases the expression of PD-L1 in osteosarcoma cells^36^. These data suggest that apatinib may be used both as a targeted therapy and as a modulator of immunotherapy in sarcoma patients.

Although the sample size was small and lacked a control group, and the follow-up time was short, our study has several merits. First, we performed the first evaluation of the efficacy and safety of apatinib in patients with stage IV osteogenic sarcoma who did not respond to chemotherapy. According to RECIST1.1, our data showed that 6 patients had SD, and the DCR, mPFS, and mOS values were 18.18% (6/33), 7.89 months, and 17.61 months, respectively. These results suggest that the efficacy of apatinib may be comparable to that of other single-agent angiogenesis inhibitors in the treatment of osteosarcoma, such as pazopanib, regorafenib, and sorafenib^11,12,17^. Second, the study indicated that apatinib had good short-term effects on osteogenic sarcoma, and 96.97% (32/33) of the patients initially responded to apatinib monotherapy. Although no patients achieved CR, and only 2 patients were evaluated as having PR, 24 patients achieved SD, and only 21.21% (7/33) had PD at 12 weeks. In a phase II clinical trial of apatinib, the researchers have analyzed data from 37 patients with progressive osteosarcoma who did not respond to chemotherapy and discovered that the fourth-month ORR and PFR, and the mPFS and mOS were 43.24% (16/37), 56.76%, 4.50 months, and 9.87 months, respectively^13^. Their results suggested that apatinib has a very high objective response rate and very good short-term effects in the treatment of osteosarcoma, but perplexingly, the DCR, PFS, and OS values were not improved. Another important original report of a randomized double-blind phase II study of regorafenib in patients with metastatic osteosarcoma has shown a significantly improved median PFS with regorafenib *vs.* placebo: 3.6 months (95% CI: 2.0–7.6) *vs.* 1.7 months (95% CI: 1.2–1.8) (hazard ratio, 0.42; 95% CI: 0.21–0.85; *P* = 0.017). In the context of the crossover design, there was no statistically significant difference in OS^12^. Some case reports had previously shown PR in patients with extraosseous Ewing’s sarcoma treated with pazopanib^37,38^. In our study on osteosarcoma, apatinib showed similar results. Consequently, although apatinib has effects in the treatment of metastatic osteogenic sarcoma, randomized clinical studies with larger sample sizes and longer follow-up time must be conducted to further clarify its therapeutic efficacy and molecular mechanism.

Common AEs to targeted anti-angiogenic drugs include hand-foot syndrome, hypertension, proteinuria, rash, diarrhea, hyperbilirubinemia, rash/desquamation, fatigue, thrombocytopenia, leukopenia, diarrhea, nausea, and vomiting^39^. In this study, the most common AEs associated with apatinib were hand-foot syndrome, proteinuria, and hypertension, which are similar to those reported in a phase III study on apatinib in patients with metastatic gastric cancer and a retrospective study in sarcoma patients^19,27^. In this study, there were no grade 4 AEs and only 2 cases of grade 3 AEs. The AEs in most patients were of grade 1 or 2 and were mainly hand-foot syndrome, proteinuria, hypertension, mucositis, fatigue, anemia, transaminase increase, and hiccups. The frequencies of these AEs in this study were similar to those in other data on sarcomas^26,27,29,30^. Hypertension was well controlled by angiotensin receptor blockers (such as valsartan) and calcium antagonists (such as amlodipine) in addition to dosage discontinuation or reduction. Hematologic AEs, including neutropenia and thrombocytopenia, were mild to moderate and did not require dosage discontinuation or reduction during treatment. These results suggest that apatinib is well tolerated, but close monitoring of treatment is still required.

## Conclusions

In summary, apatinib exhibits encouraging objective efficacy, controllable toxicity, and good short-term effects in patients with stage IV osteogenic sarcoma who do not respond to chemotherapy, but the long-term results have not been as promising as expected. Therefore, we changed the apatinib monotherapy to combined therapy and performed new clinical trials featuring apatinib in combination with chemotherapy and apatinib in combination with immunotherapy for advanced sarcomas (NCT04126993 and NCT04126811, respectively).

## Figures and Tables

**Table 1:** Recent clinical trial for advanced osteosarcoma or bone sarcoma

	Year	Cases	mPFS (month)	mOS (month)	ORR	DCR
Pazopanib^10^	2019	18	–	–	0	27.8% (7/18)
Regorafenib^11^	2019	22	3.6	11.1	13.6% (3/22)	–
Apatinib^12^	2019	37	4.5	9.9	43.2% (16/37)	–
Lenvatinib^13^	2018	26	3.4	–	7.7% (2/26)	–
Cabozantinib^14^	2018	42	6.2	–	11.9% (5/42)	–
Apatinib^15^	2018	10	7.5	14	20.0% (2/10)	70.0% (7/10)
Sorafenib^16^	2012	35	4	7	8.6% (3/35)	48.6% (17/35)

mPFS, median progression-free survival; mOS, median overall survival; ORR, objective response rate; DCR, disease control rate.

**Table 2:** Clinical characteristics of stage IV osteogenic sarcoma patients who did not respond to chemotherapy and were treated with apatinib

Characteristics	Value
Gender	
Male	23/34 (67.6%)
Female	11/34 (32.4%)
Age, years	
Average	35.24
Range	11–73
Distribution	
≤ 42	21/34 (61.8%)
> 42	13/34 (38.2%)
ECOG score	
0	2/34 (5.9%)
1	17/34 (50%)
2	14/34 (41.2%)
3	1/34 (2.9%)
Subtype (%)	
Osteosarcoma	18/34 (52.9%)
EWS/PNET	7/34 (20.6%)
Bone UPS	4/34 (11.8%)
Chondrosarcoma	3/34 (8.8%)
Chordoma	2/34 (5.9%)
Metastatic site	
Lung	34/34 (100%)
Lung and others	8/34 (23.5%)

ECOG, Eastern Cooperative Oncology Group; EWS/PNET, Ewing’s sarcoma/primitive neuroectodermal tumor; UPS, undifferentiated pleomorphic sarcoma.

**Table 3:** Efficacy of apatinib for stage IV osteogenic sarcoma that did not respond to chemotherapy

Outcome	12-week efficacy analysis*	Final efficacy analysis**
CR	0	0
PR	2	0
Osteosarcoma	1	0
Bone UPS	1	0
SD	24	6
Osteosarcoma	14	3
EWS/PNET	5	1
Bone UPS	2	2
Chondrosarcoma	1	0
Chordoma	2	0
PD	7	27
Osteosarcoma	3	15
EWS/PNET	2	6
Bone UPS	1	2
Chondrosarcoma	1	2
Chordoma	0	2
Excluded case	1 (chondrosarcoma)	1 (chondrosarcoma)
ORR	6.06% (2/33)	0
DCR	78.79% (26/33)	18.18% (6/33)
	PFR_12W_ = 82%	mPFS = 7.89 m
	OSR_12W_ = 100%	mOS = 17.61 m

CR, complete response; PR, partial response; SD, stable disease; PD, progressive disease; ORR, objective response rate; DCR, disease control rate; PFR, progression free survival rate; mPFS, median progression-free survival; OSR, overall survival rate; mOS, median overall survival. *Fisher’s exact test = 0.761, **Fisher’s exact test = 0.589.

**Table 4:** Adverse events of apatinib in the treatment of stage IV osteogenic sarcoma that did not respond to chemotherapy

Adverse event*	Grade 1	Grade 2	Grade 3	Total
Hand-foot syndrome	8	4	0	12 (35.29%)
Proteinuria	9	1	1	11 (32.35%)
Hypertension	8	3	0	11 (32.35%)
Mucositis	3	1	0	4 (11.76%)
Fatigue	3	1	0	4 (11.76%)
Anemia	4	0	0	4 (11.76%)
Transaminase increase	4	0	0	4 (11.76%)
Hiccups	4	0	0	4 (11.76%)
Pain	0	3	0	3 (8.82%)
Diarrhea	3	0	0	3 (8.82%)
Skin pigmentation	3	0	0	3 (8.82%)
Periodontal disease	3	0	0	3 (8.82%)
Rash	3	0	0	3 (8.82%)
Bilirubin increase	3	0	0	3 (8.82%)
Pneumothorax	2	0	0	2 (5.88%)
Dysgeusia	0	0	1	1 (2.94%)
Apositia	0	1	0	1 (2.94%)
Hematuria	1	0	0	1 (2.94%)
Dyspnea	1	0	0	1 (2.94%)
Total	62	14	2	78

*According to CTCAE5.0.
